# Cultural turnover among Galápagos sperm whales

**DOI:** 10.1098/rsos.160615

**Published:** 2016-10-19

**Authors:** Mauricio Cantor, Hal Whitehead, Shane Gero, Luke Rendell

**Affiliations:** 1 Department of Biology, Dalhousie University, Halifax, Canada; 2 Zoophysiology, Department of Bioscience, Aarhus University, Aarhus, Denmark; 3 School of Biology, University of St. Andrews, St Andrews, UK

**Keywords:** culture, dialect, *Physeter macrocephalus*, population ecology, demographic change, social structure

## Abstract

While populations may wax and wane, it is rare for an entire population to be replaced by a completely different set of individuals. We document the large-scale relocation of cultural groups of sperm whale off the Galápagos Islands, in which two sympatric vocal clans were entirely replaced by two different ones. Between 1985 and 1999, whales from two clans (called *Regular* and *Plus-One*) defined by cultural dialects in coda vocalizations were repeatedly photo-identified off Galápagos. Their occurrence in the area declined through the 1990s; by 2000, none remained. We reassessed Galápagos sperm whales in 2013–2014, identifying 463 new females. However, re-sighting rates were low, with no matches with the Galápagos 1985–1999 population, suggesting an eastward shift to coastal areas. Their vocal repertoires matched those of two other clans (called *Short* and *Four-Plus*) found across the Pacific but previously rare or absent around Galápagos. The mechanisms behind this cultural turnover may include large-scale environmental regime shifts favouring clan-specific foraging strategies, and a response to heavy whaling in the region involving redistribution of surviving whales into high-quality habitats. The fall and rise of sperm whale cultures off Galápagos reflect the structuring of the Pacific population into large, enduring clans with dynamic ranges. Long-lasting clan membership illustrates how culture can be bound up in the structure and dynamics of animal populations and so how tracking cultural traits can reveal large-scale population shifts.

## 1. Introduction

Behavioural repertoires change over time. Changes may result from adaptive genetic evolution and genetic drift, phenotypic plasticity, and individual or social learning (e.g. [1,2]). Changes can take many generations (typical for genetic evolution) or occur over a small part of the life cycle (e.g. phenotypic plasticity in the face of rapid environmental change). When behaviour is socially learned and shared, hence culture, evolutionary processes influence these dynamics at various scales [2,3]. When cultural behaviour changes rapidly relative to generation time, it can do so in two distinct ways. First, by replacement of behaviours: individuals learn new behaviours and those spread through the standing population. Second, by replacement of the individuals themselves: the population using that area dramatically changes in composition such that others replace the entire cultural trait groups.

There are multiple non-human examples of the first case—replacement of behaviours. Male humpback whales (*Megaptera novaeangliae*) sing a continuously evolving population-specific song [4], but in the South Pacific, populations discard entire songs in favour of a new song from a neighbouring population in a revolutionary transition that takes less than a year [5,6]. Similarly, humpback populations can rapidly diffuse foraging innovations [7]. As for the second case—replacement of individuals—there are examples from human history of cultural groups replacing each other in a given territory. One is the history of the Sahel, the sub-Saharan semiarid vegetation belt that was once home for different cultural groups with two distinct feeding strategies, nomadic pastoralism and sedentary farming [8]. Following large-scale environmental changes after the French colonial rule (a combination of natural and anthropogenic desertification), groups whose feeding strategies no longer fit the habitat were forced to move [8,9], resulting in a cultural turnover caused by the replacement of individuals by those from different culturally defined groups. However, examples outside humans are much rarer or non-existent. Here, we document rapid cultural turnover in an animal population caused by the replacement of cultural groups on an oceanic scale: the sperm whales (*Physeter macrocephalus*) off the Galápagos Islands.

Female sperm whales live in multilevel societies [10]. The fundamental social level is the nearly permanent social units of about 11 females and their young [10–12]. The largest level is the vocal clan, that we distinguish using characteristic repertoires of codas, i.e. stereotyped patterns of broadband clicks used in social communication [13]. Vocal clans are sympatric [14] but socially segregated. Social units of a clan only form temporary groups (about 2–3 social units typically over periods of days) with other units of the same clan, i.e. that share the same repertoires of coda types united by a common structural theme [14]. Two vocal clans were common around the Galápagos Islands in the 1980s–1990s: the *Regular* clan, consisting of social units that mostly make codas with regularly spaced clicks and the *Plus-One* clan, most of whose codas have an extended interval before the last click. Two other clans were identified across the wider Tropical Pacific: the *Short* clan, which mostly produced brief codas with fewer than five clicks; and the *Four-Plus* clan, which mostly produced codas with a base of four regular clicks [14]. These distinct coda dialects are stable over at least a decade [15]. Among clans, there is extensive sharing of mitochondrial DNA haplotypes, thus, taken with the degree of sympatry, it is almost certain that these dialect variations are cultural in nature [16]. Clans also differ in habitat use, foraging success, diet, social behaviour and possibly calving rates [17–19], suggesting that clan membership has much wider implications than just vocal dialect. Thus, clans appear to be a significant structuring factor in sperm whale society.

Although highly socially structured, sperm whales display little geographical structure: clans overlap over very large areas [10,14]. While social units have ranges spanning about 2000 km, the clans to which they belong have wider distributions, spanning across the Tropical Pacific [14,20]. This nomadic behaviour probably reflects adaptive space use, probably driven by the effects of oceanographic conditions on variation in the distribution of their prey, deep ocean squid [20,21]. We surveyed sperm whale populations and coda repertoires over the last three decades, and use these data here to show a complete turnover in cultural dialects concurrent with a turnover in the pool of individuals around the Galápagos Islands.

## 2. Material and methods

### 2.1. Field methods, photo-identification and acoustic recordings

**Figure 1.**
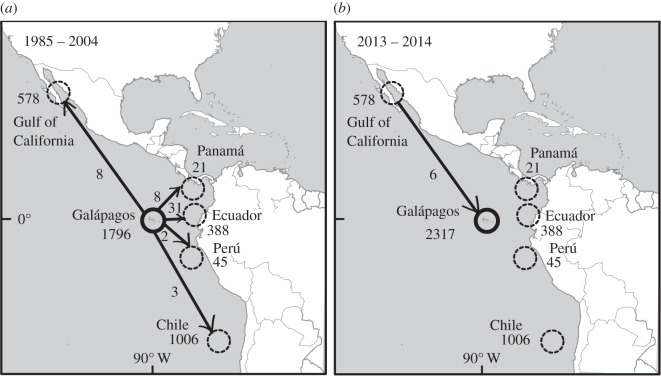
Re-sightings of individual female and immature sperm whales between (*a*) 1985 and 2004 in the eastern Tropical Pacific, and (*b*) 2013 and 2014 off Galápagos. Dashed circles loosely indicate study areas, with numbers indicating total of photo-identified individuals. Numbers by arrows indicate match of individuals between areas.

Sperm whales were tracked visually and acoustically in deep waters (more than 1000 m) across the Tropical Pacific, day and night during two- to four-week surveys between 1985 and 2014 (figure 1; electronic supplementary material, tables S1–S3). Given the logistical challenges of offshore surveys, sampling was unevenly distributed; the Galápagos archipelago was the main study area (electronic supplementary material, tables S1–S3). Annual encounter rates off the Galápagos were calculated as number of groups of female and immature whales encountered divided by total hours of acoustic and visual search (i.e. total effort minus time following whales) [22].

Individuals were identified from photographs based on patterns of natural marks on the trailing edge of their tails, assisted by a semi-automated photo-identification protocol [23]. We rated each photograph from poor to very high quality (*Q* = 1–5) based on focus, exposure, orientation, per cent cover and tilt of the fluke [24]. Distinctively small animals (of about less than 2 years) were considered calves; distinctively large animals as mature males; the others were considered females and immatures [24]. We analysed only *Q* ≥ 3 photographs of females and immatures. From a total of 14 286 photographs, we identified 4468 individuals (electronic supplementary material, table S2).

Sperm whale codas were recorded using various hydrophone arrays and recording devices over the duration of the study (electronic supplementary material, method S1) [14]. All recordings were analysed using Rainbow Click software [25] in which individual clicks were manually marked and designated as part of codas. From acoustic recordings from across the Pacific, we sampled 17 045 codas (electronic supplementary material, table S3).

### 2.2. Assigning coda repertoires to photo-identified groups

We assigned coda recordings to groups of individual whales photo-identified together. We considered all recordings made on the same day when continuously following a group of sperm whales to be of the same group [14]. Codas recorded on two different days were considered to be from the same group if at least 25% of the photo-identified individuals were re-sighted [26]: *m_ab_* > 0.25* × *min{*n_a_, n_b_*}, where *m_ab_* is the number of individuals photo-identified on both days, *n_a_* is the number of individuals identified on the first day, and *n_b_* on the second day. We discarded groups whose recorded repertoires contained less than 25 codas [14].

To account for any potential autocorrelation in coda production during the same day, all coda recordings on a given day from a given group represented a single repertoire. Under the assumption that coda production of a given group on a given day is independent of its production on a subsequent day, repertoires from different days were treated as replicates of a group's repertoire and were considered independent samples of a group's coda production [27]. We used permutations to test differences between group repertoires (electronic supplementary material, method S2).

### 2.3. Continuous and categorical similarity between coda repertoires

We compared group repertoires using the absolute inter-click intervals (ICIs, i.e. the time between the onset of one click to another in a coda sequence) to represent the temporal structure (rhythm and tempo) of their codas [27,28]. To quantify similarity between coda repertoires, we applied continuous and categorical metrics to this multivariate dataset. The former was used to define the vocal clan partitions; the latter was used to define coda types that illustrate the differences in the patterning theme of the codas between clans (analyses pathway: electronic supplementary material, figure S1).

For the continuous approach, we calculated the multivariate similarity of two codas of the same click length (i.e. same number of clicks) using the Euclidean distance between their ICI vectors (electronic supplementary material, method S3) [14]. With the categorical approach, we classified codas into discrete types based on their rhythm and tempo using OPTICSxi hierarchical clustering [29] (in this context: [27]). We ran OPTICSxi on the absolute ICI independently for each set of codas of the same click length, performing a sensitivity analysis *a priori* to define the algorithm initial parameters (electronic supplementary material, method S4). We labelled the coda types according to number of clicks and rhythm, based on previous nomenclature [14,26].

### 2.4. Assigning photo-identified groups to vocal clans

The original partition of vocal repertoires into clans [14] (electronic supplementary material, method S5) used hierarchical clustering analyses based on the continuous multivariate similarities of standardized ICIs of codas, and the *k*-means algorithm to categorize codas into types [14]. Here, we used the updated methods for comparing repertoires described above to re-analyse this data set together with the repertoires recorded off Galápagos in 2013 and 2014 (electronic supplementary material, table S3). To assign the 2013 and 2014 groups to clans, we first built an average-linkage clustering dendrogram using the continuous multivariate similarity matrix for the combined dataset; then we identified whether these groups clustered together into a distinctive branch (indicating a new clan) or whether they clustered with previous clans. We measured the accuracy of the dendrogram representation using the cophenetic correlation coefficient (CCC), and considered CCC > 0.8 to indicate a reliable representation. The dendrogram robustness was measured by bootstrap resampling [14]. All groups' coda repertoires were randomly sampled with replacement (100 replicates), their similarities were recalculated and the proportion of times a given branch was replicated used to indicate the robustness of that branch.

## 3. Results

### 3.1. Photographic matching and movements

**Figure 2.**
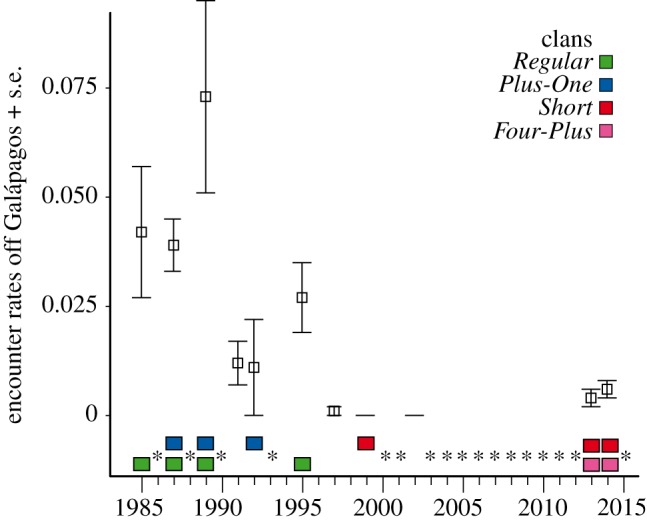
Encounter rates of sperm whale off the Galápagos Islands over 30 years across clans. Rates were higher in early 1980s, started declining during the 1990s and, after a hiatus in 2000s, started rising again. Colour code indicates clan membership (figure 3) of whales for which both photo-identification and acoustic data were available in that year. Whiskers represent standard errors (s.e.). Asterisks indicate years with no dedicated surveys off Galápagos (the larger gap in the 2000s was mainly motivated by a lack of opportunistic sightings in the area), but in some of these years there were surveys in surrounding areas (electronic supplementary material, tables S1–S3).

We identified 4468 individuals across the Pacific study areas (electronic supplementary material, table S2) with re-sightings illustrating the scale of movements individuals could undertake (figure 1). Most individuals were identified off the Galápagos Islands; however, overall encounter rates there declined over the period 1985–2000 (figure 2). Between 1985 and 1995, female and immature sperm whales were repeatedly found (1085 identified individuals); encounters with whale groups became rarer in the late 1990s and by the 2000s they had left the area (figure 2). Surveys from 1985–2004 suggested an eastward movement away from Galápagos (figure 1*a*). Our 2013–2014 surveys indicated a modest, recent return of sperm whales to this area (figures 1*b* and 2); however, this was by new individuals. The photographic recapture rate was very low: only 1% of the females and immatures (5/463) were sighted in both 2013 and 2014. From these recently photo-identified whales, none matched with the previous whales seen off Galápagos and only six females had been seen in in the Gulf of California in 2003 (figure 1*b*).

### 3.2. Acoustic repertoires

From 1985 to 1999, coda repertoires of 64 groups of female and immature were recorded across the Tropical Pacific [14]; in 2013–2014, we recorded 15 new groups off the Galápagos (electronic supplementary material, table S3). Mantel tests confirmed that repertoire similarity between acoustic recordings from the same group was greater than between different groups indicating that groups had significantly different repertoires (Galápagos 2013–2014: *r* = 0.413, *p* < 0.001; Pacific 1985–1999: *r* = 0.170, *p* < 0.001; combined: *r* = 0.176, *p* < 0.001).

**Figure 3.**
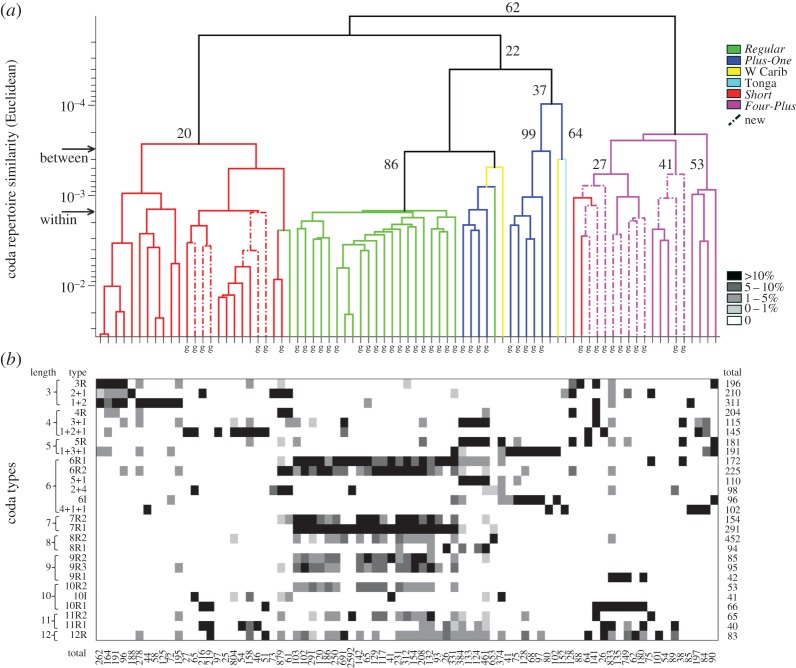
Coda repertoire similarity and clan structure of sperm whale groups from the Pacific between 1985 and 2014. (*a*) Hierarchical clustering dendrogram (CCC = 0.896) depicts the multivariate similarity (Euclidean distances on absolute inter-click intervals) among coda repertoires of photo-identified groups of sperm whales (branches). Colour code and clan names follow original results [14]; ‘g’s indicate groups observed off Galápagos; dashed branches indicate new groups observed in 2013–2014; arrows indicate mean similarity between and within clans; numbers besides nodes indicate the number of replications (out of 100) in bootstrap analysis. (*b*) Frequency of coda types containing up to 12 clicks (rows) classified into discrete types for each photo-identified group (columns). Shades of grey indicate the frequency of occurrence of coda types in a given group repertoire; coda type labels represent rhythm (electronic supplementary material, figure S2); numbers under columns indicate total number of recorded codas from each group of whale used in the continuous analysis; numbers on the right indicate the total codas per type used in the categorical analysis.

The categorical analysis of the full dataset identified 27 distinct coda types containing from 3 to 12 clicks varying in rhythm and tempo (electronic supplementary material, figure S2). Although the OPTICSxi algorithm classified only the most stereotyped codas (4091/17 045 codas; 24%), discovery curves of classified coda types were nearly asymptotic (electronic supplementary material, figure S3), suggesting that most coda types made by the sampled groups were represented. Coda types were robust to variation across the OPTICSxi input parameter space (electronic supplementary material, figure S4). Coda type classification described the thematic differences in coda patterning driving clan partitioning, defined by the continuous analysis including all codas (figure 3).

### 3.3. Clan structure

The original partitioning of clans in the Tropical Pacific [14] was preserved in our analysis, with *Regular*, *Four-Plus* and *Short* clans depicted in our dendrogram as largely similar to the original analysis (figure 3*a*). There were some minor changes: the groups recorded off Tonga and in the western Caribbean (branches with lower bootstrap support in the original analysis) clustered with groups belonging to the *Plus-One* clan; and four groups (two designated as *Short*, and two as *Regular*) clustered with different clan branches (figure 3*a*). We expected some changes because our new analysis was different from the original [14] in two ways. First, we included the newer groups recorded off Galápagos (electronic supplementary material, figure S5). Second, we used absolute instead of the relative ICI used in the original clan partition, as recent studies [27,28] suggest that tempo, in addition to rhythm, is an important element of coda diversity. Nevertheless, the dendrogram in figure 3 is an appropriate depiction of the coda repertoire similarity among groups of whales (CCC = 0.896), with good support from the bootstrap analysis.

The new categorical coda classification reproduced the main thematic patterning expected for coda types in each clan (figure 3*b*). For instance, groups belonging to the *Regular* clan mainly produced regularly spaced codas from 6 to 12 clicks (e.g. 6R1, 6R2, 7R1, 7R2, 8R2, 9R2, 9R3, 10R2, 11R, 12R); groups from the *Short* clan mainly produced codas with 3 to 5 clicks (e.g. 3R, 2 + 1, 1 + 2, 4R, 1 + 2 + 1, 1 + 3 + 1); *Plus-One* groups produced mainly codas with an extended pause before the last click (e.g. 3 + 1, 1 + 3 + 1, 5 + 1, 4 + 1 + 1); *Four-Plus* groups produced codas with four regular clicks (e.g. 4R, 4 + 1 + 1). The groups recorded off Tonga and in the western Caribbean contained dominant codas with longer pauses at the end (e.g. Tonga: 4 + 1 + 1; Caribbean: 1 + 3 + 1, 5 + 1, 6I, 10I). These coda types may explain the tendency for Tonga and Caribbean groups to cluster with the *Plus-One* clan in our analysis. The patterns seen in the clustering analysis agreed with the distribution of coda types per clan in the multivariate space: some types were made by many clans, whereas other types were characteristic of some clans as described above (electronic supplementary material, figures S6 and S7).

The repertoires of groups recorded in 2013 and 2014 off Galápagos did not cluster by year of recording (electronic supplementary material, figure S5), indicating that different clans were present in both years. When these new groups were added into the Tropical Pacific clan analysis, they clustered with existing branches representing the *Four-Plus* and *Short* clans and not with the *Regular* and *Plus-One* clans previously heard off Galápagos (figures 2 and 3*a*). *Four-Plus* and *Short* were heard previously off Chile, Kiribati and the Marshall Islands, and were very rare or absent off Galápagos in the past: in fact, only a single social unit of the *Short* clan was recorded in 1999 (figure 2) [14]. Our acoustic results concurred with the photo-identification results: the lack of matches between the Galápagos whales from 1985–1999 and 2013–2014 (electronic supplementary material, table S2); and the six whales seen in 2003 in the Gulf of California (figure 1*b*) were found to be members of the *Four-Plus* clan in 2013 off Galápagos (figure 3*a*).

## 4. Discussion

Our study demonstrates cultural turnover in the sperm whale dialects off the Galápagos Islands over the last 30 years. We attribute these changes to a turnover in the clans utilizing these waters, as the shift in the acoustic repertoires matches the complete replacement of individual sperm whales off the Galápagos. These findings confirm previous suggestions that clans are stable over time (at least in repertoire, and almost certainly in membership) but dynamic over space [15]. Our long-term analysis indicates that the coda repertoires remain little changed across three decades in the Pacific, highlighting that Pacific sperm whales roam over very wide geographical areas as members of large, long-lasting cultural clans [14,30].

None of the individual sperm whales using the waters off Galápagos in 2013 and 2014 were seen in the area during the previous three decades. Our photo-identification findings increased the number of individual sperm whales catalogued across the Tropical Pacific, but the rate of photographic recaptures off the Galápagos between 2013 and 2014 was low despite our long-term and large-scale sampling. Offshore surveys, however, impose several logistical challenges making our sampling effort patchy in time and space (electronic supplementary material, table S1). We acknowledge the consequent uncertainty regarding presence of whales in waters near the Galápagos, as well as in unsampled years. Yet, our photo-identification data provide strong evidence for large-scale movements between discrete study areas and across years. We propose, therefore, that the drastic demographic change we report was driven by emigration of groups of whales from different clans.

There are four lines of evidence that support emigration out of Galápagos, rather than changes in the composition of the clans themselves, as the most likely mechanism for the local decline in sperm whale sightings. First, there were several re-identifications of Galápagos groups and clans off northern Chile and the Gulf of California (figure 1*a*, see also [20,31]), evidencing that sperm whales do move long distances. Second, sperm whales seem not change their clan membership, or if so, only very rarely [24]. Third, sperm whales are slow-reproducing, long-living animals [24] and the last three decades is a relatively short window in their lifespan during which no high mortality was evident [22]; all of these make death and birth very unlikely to be the drivers of the replacement of individuals off Galápagos. Finally, errors in individual identification cannot be a major factor because marks used to photo-identify animals rarely change [32]; indeed, Atlantic sperm whale individuals have been re-identified across 30 years within a single study area much smaller than ours [12]. Combining these facts with our findings on coda repertoires, we suggest that the same clans from three decades ago still populate the Tropical Pacific, but what seems to have occurred is a large shift in the habitat used by each of the clans.

Sperm whales are nomadic. In the Pacific, social units have wide ranges, performing long-distance movements (mostly spanning about 2000 km, some over 4000 km) within relatively short temporal scales [20], emphasizing the magnitude of the spatial scale relevant for sperm whales. The long-distance movements are made by individuals travelling together, because sperm whales live in nearly permanent social units [11]. These units belong to large clans with dynamic ranges [10,15], which are stable emergent social structures [30] within which coda usage is conserved over time [15]. Therefore, the radical cultural turnover in sperm whale dialects off Galápagos reflected a clan replacement, i.e. a local turnover in whales using the area as a consequence of their natural movements over large spatio-temporal scales. This contrasts with the cultural revolutions among humpback whales where songs changed dramatically but with little turnover of individuals [4–6].

The sperm whales recently identified off Galápagos are not members from the *Regular* and *Plus-One* clans once common in the area. Instead, they are members of two existing clans (*Four-Plus* and *Short*) previously heard across the Pacific but very rare or absent in Galápagos waters. The *Four-Plus* clan was consistently heard off northern Chile, while the *Short* clan spread over the Tropical Pacific and only few of its members (a single social unit) had been previously identified off Galápagos [14]. The new whales immigrated from neighbouring waters in the wider Pacific. Our photo-identification data may give some indication of origin: there were some matches with *Four-Plus* clan members seen previously in the Gulf of California, where both *Short* and *Four-Plus* clans may be present [33].

### 4.1. Why were the clans replaced?

The Galápagos Islands, and more broadly the eastern Pacific, were historically important grounds for sperm whales [34]. Although there were numerous whales when our Galápagos studies started in 1985, emigration drastically reduced their numbers between 1990 and 2000. Following this exodus, members of different clans have been slowly repopulating the Galápagos. The fall and rise of sperm whale clans off Galápagos lead to two questions. Why did members of the original clans leave? Why are the new whales from other clans rather than return of the original clans? While our data show a clear shift in Galápagos sperm whale dialects, the underlying mechanisms for the large-scale displacement of clans are necessarily speculative. In what follows, we describe two non-exclusive hypotheses.

The first scenario involves large-scale environmental shifts. Drastic environmental changes force cultural groups to adapt their strategies or move (e.g. [8]). Like many other predators as well as some herbivores, food availability is a major driver of movement for sperm whales. They tend to go where the prey is, moving from areas of low to high feeding success [35]. The abundance of preferred prey, for instance jumbo squid (*Dosidicus gigas* [21]), may fluctuate naturally across the Pacific and in response to environmental changes such as the El Niño Southern Oscillation (ENSO). ENSO events impose massive changes in the Pacific [36], including anomalous sea surface warming, large influx of deep warm waters, and fluctuations in primary productivity and nutrient cycling [36,37]. In particular, the extreme ENSO events in the early 1980s and late 1990s represented remarkable warming in the equatorial Pacific, devastating marine fauna [36,38], including marine communities in the Galápagos region [39]. The reduced productivity of tropical and equatorial Pacific waters considerably decreased the feeding success of sperm whales off Galápagos [40]. ENSO events are becoming more frequent and intense [36,41]; due to cetaceans' high and adaptive mobility, leaving affected areas is their immediate response [42]. We know sperm whales from different clans tend to move and forage differently [17]. In years of normal temperatures, the foraging strategy of the *Regular* clan outperforms the *Plus-One*; whereas in the warmer, less productive ENSO years the foraging successes of both clans is reduced considerably but the *Plus-One*'s strategy becomes more efficient than the *Regular*'s [17]. Clans may conserve their foraging strategies even during remarkable environmental changes [17], thus living in this large-scale dynamic habitat, groups of whales from particular clans may relocate, moving to areas where their foraging strategies are likely to maximize their food intake. This assumed cultural inertia of foraging strategy—not uncommon in marine mammals [43,44]—implies that large-scale movement is favoured over remaining in a changing habitat and adapting to the new conditions. This may explain both why the original clans left and why the new immigrants are from different clans, but implies that changes to the ecosystem around Galápagos [37–39] are perceived differently by sperm whales from different clans.

The second scenario involves lagged responses to the population decline caused by modern whaling [22]. Sperm whales in the general vicinity of the Galápagos were heavily hit by nearly unregulated, as well as pirate, whaling between 1957 and 1981 [45,46]. The extreme depletion of sperm whales of the eastern Pacific in those years focused on the relatively inshore waters of the Humboldt Current off Peru and Chile as the legal whaling used catcher-boats operating from mainland ports [34,46]. The whaling may have left a surplus of sperm whale prey, re-opening a niche in the rich Humboldt Current waters. In the case of density-dependent habitat selection [47], whales would redistribute themselves according to habitat quality. Therefore, low whale density in productive coastal waters may have stimulated the eastward migration out of the Galápagos in the 1990s [22]. If the population slowly recovers, it would redistribute to first occupy high-quality coastal waters then adjacent areas [47], which may explain the modest and recent return to Galápagos waters documented here. In this scenario, the turnover of clans off the Galápagos would result from a general eastward movement: first of the *Regular* and *Plus-One* clans from the Galápagos to more coastal waters, and then of the *Four-Plus* and *Short* clans from oceanic and northern waters to the Galápagos. The underlying assumption is that different cultural foraging strategies characteristic of each clan [17] perform similarly in different areas.

In both scenarios, the turnover of clans using the Galápagos indicates that there may be social dynamics driving movement decisions. Group displacement implies a compromise between individual decision and group conformity (e.g. [48]). Thus, once some members of one clan have decided to leave a particular habitat, other members may choose to move with clan-mates rather than remain within that habitat—a within-clan gregariousness that may be mediated by specific codas that identify clan membership [27]. As associations with familiar conspecifics can facilitate acclimation to novel habitat [49], the benefits of foraging and associating with behaviourally similar clan members may outweigh the cost of displacement to a new habitat. This assumes that clan membership is important for the success of the individuals and social units that comprise them, which fits well with recent evidence that sperm whale movement decisions are shared [50] and that individuals conform to the predominant behaviour of clan members [30]. Overall, these findings show that tracking cultural traits can reveal large-scale population shifts, which further illustrates the key role culture can play in the structure and dynamics of animal populations and their communication systems.

## 5. Conclusion

Learned communication repertoires can be either stable across or change within generations in response to cultural selection and drift [51–53]; yet a population's repertoire is rarely completely replaced. We found an influx of immigrants from different cultural clans replacing those that used to be in the area decades ago. This local cultural turnover was an epiphenomenon of large-scale displacement of sperm whales organized by vocal clan, suggesting that clan structure is temporally stable but spatially flexible. The changes in sperm whale acoustic repertoires off Galápagos are clear, but the ultimate causes of this cultural turnover remain unclear. Unravelling the drivers of large-scale relocation of cultural groups will allow us to better understand animals' response to the changing ocean, the dynamics of depressed populations and the importance of culture in animal societies.

## Supplementary Material

We submit a single PDF file with supplementary data in 3 tables, supplementary results in 7 figures, and methods details.

## Data accessibility

All data to reproduce the results are deposited at the Dryad repository: http://dx.doi.org/10.5061/dryad.8jj26 [54].
