# Supplementary material for: Cultural turnover among Galápagos sperm whales
Source: R Soc Open Sci. 2016 Oct 19;3(10):160615. doi: 10.1098/rsos.160615 (PMC5099007; doi:10.1098/rsos.160615)
Supplement: We submit a single PDF file with supplementary data in 3 tables, supplementary results in 7 figures, and methods details. [file rsos160615supp1.pdf]

Supplementary Material: Cultural turnover among Galápagos sperm whales

Mauricio Cantor*, Hal Whitehead, Shane Gero, Luke Rendell

* Correspondence: m.cantor@ymail.com

Table S1: Sampling effort summary of dedicated visual and acoustic surveys for sperm whales in the Eastern Tropical Pacific (ETP). Study areas: Galápagos Islands (1), Panamá, Ecuador, and northern Peru (2), southern Peru and Chile (3), Gulf of California (4) and Western Pacific (5). Days following whales: total days tracking and in visual contact with sperm whale groups; Photographic records: total number of pictures taken of tail flukes for photo-identification (see also [1]); Codas analyzed: total number of coda samples manually identified and included in the continuous analyses (see also [2]).

Year	Study areas	Days following whales	Photographic records	Codas analyzed
1985	1,2	32	973	1,511
1987	1	55	1,615	2,342
1988	1	15	442	0
1989	1	56	1,708	113
1991	1,2	25	962	413
1992	2,4,5	13	273	1,234
1993	2,3,4,5	26	514	1,553
1994	1	3	108	0
1995	1,2,4	23	583	660
1996	1	8	144	0
1997	1,4	20	310	0
1998	1,4	27	179	0
1999	1,2,4,5	46	811	879
2000	2,3	79	2,082	5,360
2002	1,4	35	897	0
2003	4,5	17	383	0
2004	4	6	173	0
2013	1	27	996	1,986
2014	1	41	1,113	994
Total	ETP	554	14,286	17,045

In 2013 and 2014, we revisited the Galápagos waters, totaling 165 sampling days and 1,670 hours with visual and/or acoustic contact with 15 groups of distinctive whales photo-identified together**.**

Table S2: Number of photo-identified (quality rating $Q \geq 3$) individual adult females sperm whales and immatures of both sexes (males in parentheses) in the Tropical Pacific by study area and year. Only individuals with a recorded position are presented. Calves were not quantified. Data from 1985-2004 (3,947 individuals) came from [1].

Year	Galápagos	Panamá, Ecuador, Northern Perú	Chile, Southern Perú	Gulf of California	Western Pacific	Total
1985	344 (8)	6 (0)	0 (0)	0 (0)	0 (0)	358
1987	440 (8)	0 (0)	0 (0)	0 (0)	0 (0)	448
1988	95 (0)	0 (0)	0 (0)	0 (0)	0 (0)	95
1989	337 (4)	0 (0)	0 (0)	0 (0)	0 (0)	337
1991	94 (2)	278 (4)	0 (0)	0 (0)	0 (0)	378
1992	0 (0)	5 (0)	0 (0)	1 (0)	109 (5)	120
1993	0 (0)	116 (1)	114 (0)	2 (0)	5 (0)	238
1994	23 (0)	0 (0)	0 (0)	0 (0)	0 (0)	23
1995	91 (8)	21 (0)	0 (0)	4 (0)	0 (0)	124
1996	29 (4)	0 (0)	0 (0)	0 (0)	0 (0)	31
1997	0 (0)	0 (0)	0 (0)	5 (0)	0 (0)	5
1998	27 (6)	0 (0)	0 (0)	64 (4)	0 (0)	101
1999	9 (3)	3 (0)	0 (0)	102 (3)	1 (0)	120
2000	0 (0)	15 (0)	863 (32)	0 (0)	0 (0)	910
2002	0 (37)	0 (0)	0 (0)	280 (3)	0 (0)	420
2003	1 (0)	0 (0)	0 (0)	139 (4)	1 (0)	141
2004	0 (0)	0 (0)	0 (0)	68 (0)	0 (0)	68
2013	210 (25)	0 (0)	0 (0)	0 (0)	0 (0)	235
2014	253 (33)	0 (0)	0 (0)	0 (0)	0 (0)	286
Total	1,953 (138)	444 (5)	997 (32)	665 (16)	116 (5)	4,468

Table S3: Number of codas analyzed from groups of female and immature sperm whales photo-identified together in the Eastern Tropical Pacific by study area and year. Year correspond to the photo-identification effort in Table S1. Data from 1985-1999 (14,065 codas) came from [2].

Year	Galápagos	Panamá, Ecuador, Northern Perú	Chile, Southern Perú	Gulf of California	Western Pacific	Total
1985	1,511	0	0	0	0	1,511
1987	2,342	0	0	0	0	2,342
1988	0	0	0	0	0	0
1989	113	0	0	0	0	113
1991	0	413	0	0	0	413
1992	0	293	0	941	0	1,234
1993	0	429	616	134	374	1,553
1994	0	0	0	0	0	0
1995	660	0	0	0	0	660
1996	0	0	0	0	0	0
1997	0	0	0	0	0	0
1998	0	0	0	0	0	0
1999	879	0	0	0	0	879
2000	0	0	5,360	0	0	5,360
2002	0	0	0	0	0	0
2003	0	0	0	0	0	0
2004	0	0	0	0	0	0
2013	1,986	0	0	0	0	1,986
2014	994	0	0	0	0	994
Total	8,485	1,135	5,976	1,075	374	17,045

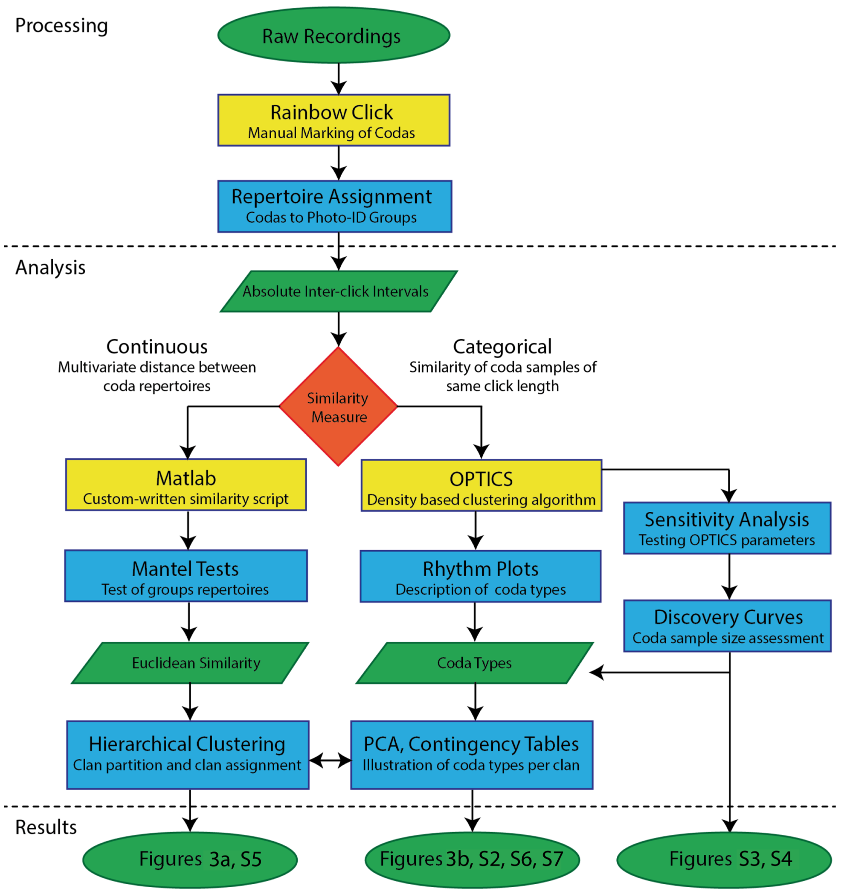

Figure S1. Schematic of the analyses of acoustic recordings and coda repertoires to define vocal clans of sperm whales. In the processing phase, raw acoustic recordings were listened at ¼ speed and codas were marked in the Rainbow Click software. Coda repertoires were composed of coda recordings assigned to groups of whales photo-identified together. In the analysis phase, we used the absolute inter-click intervals (ICI, the actual time between the onset of one click to another in a coda sequence) to characterize the temporal structure of the codas. We then used two complementary analyses, continuous and categorical. With the continuous measure, we compared coda repertoires similarity among groups of whales, using customized MATLAB routines to calculate the multivariate Euclidean distance between repertoires and hierarchical clustering algorithm (supported by a bootstrap procedure) to define the partition of groups into clans of whales (figure 3a). With the categorical measure, we qualitatively described the differences between clan repertoires. We used the OPTICS algorithm to identify clusters of very similar coda

samples, with stereotyped rhythm and tempo (figure S2), which we called coda types. We performed a sensitivity analysis to define the most parsimonious initial parameters for the OPTICS algorithm (figure S4). Many coda samples were disregarded as 'noise' and not included in a coda type cluster; however nearly asymptotic discovery curves suggested that nearly all coda types made by the sampled groups were represented (figure S3). We then used contingency tables (figure 3b) to illustrate the principal differences between the repertoires of the different clans (figure 3a), and Principal Component Analysis (figures S6, S7) to visualize with more details these differences in coda type usage by different clans. This schematic was adapted from [3].

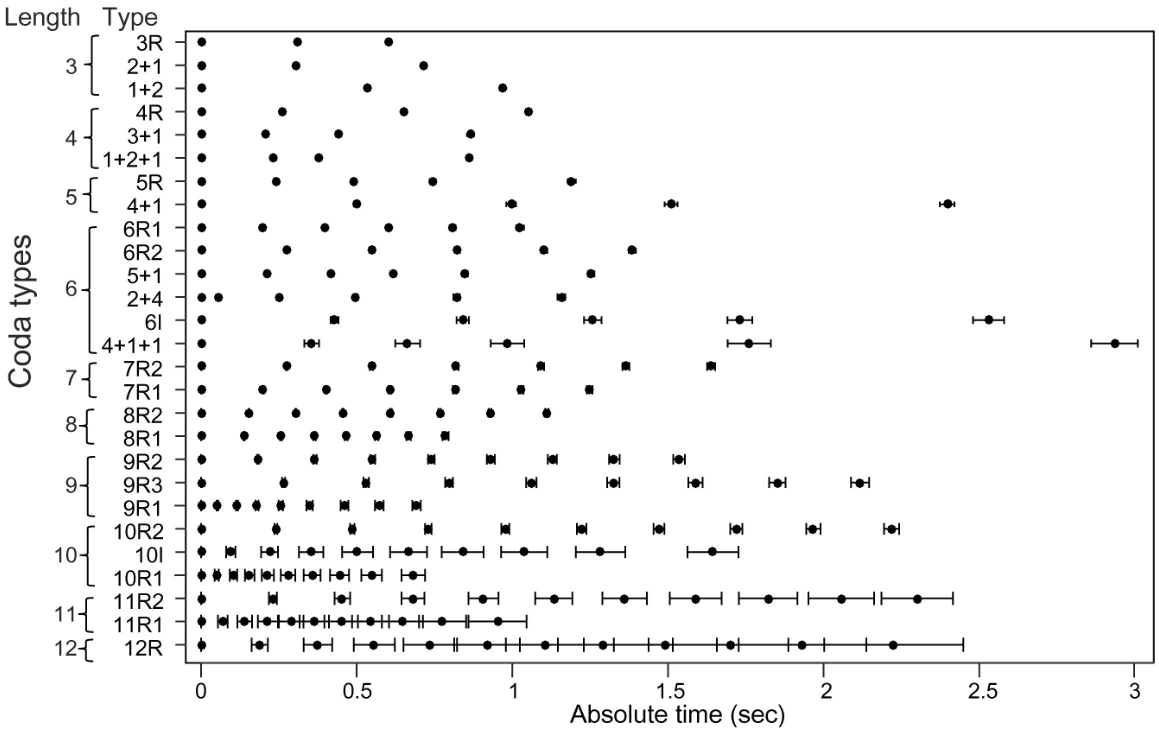

Figure S2. Codas up to 12 clicks produced by sperm whales across the Eastern Tropical Pacific and classified into discrete types. Coda types (y-axis) were defined by the OPTICS algorithm (fixed ξ=0.025 and *minpts* ~4% of the sample size) based on absolute time (x-axis) between inter-click intervals (ICIs). Dots represent mean time of each click in the coda, and whiskers represent 95% Confidence Intervals around the mean time. Coda type labels were based on the rhythm, where the first number indicates the number of clicks; "R" indicates regularly spaced clicks; "I" indicates increasing click intervals along the coda; "+" indicates extended interval between clicks; and the sequential number distinguishes between codas with the same number of clicks and rhythm but of increasing duration (e.g. 9R1, 9R2, 9R3).

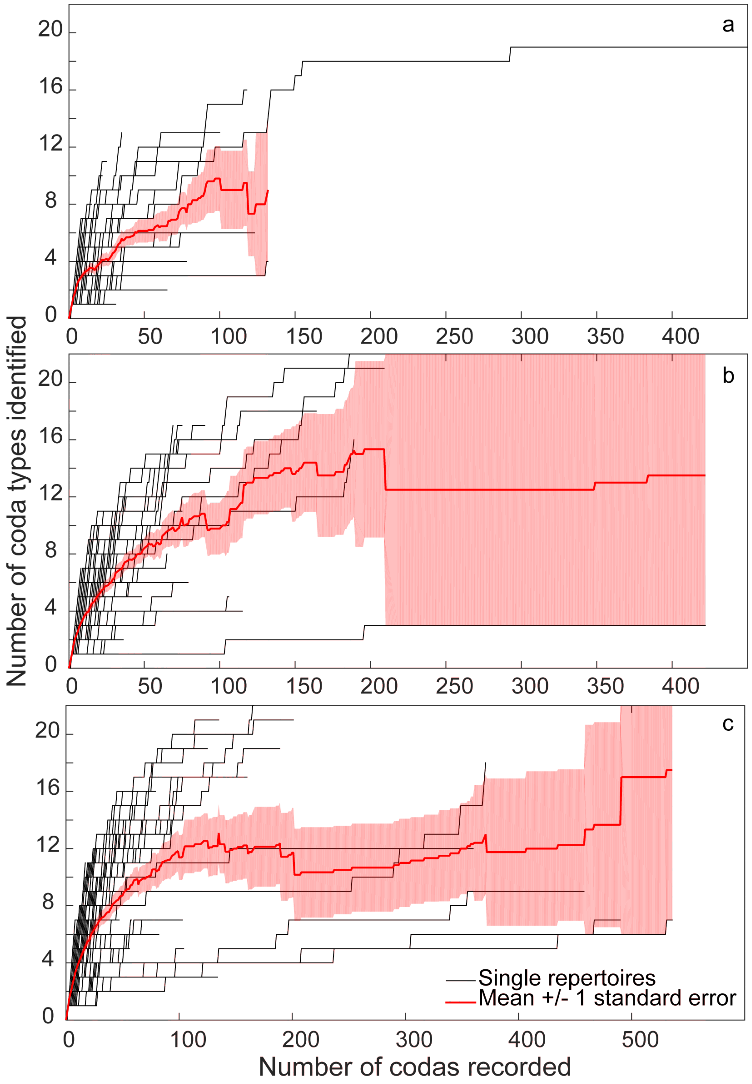

Figure S3. Discovery curves for coda types per photo-identified group of sperm whales across the Eastern Tropical Pacific. Coda types were identified using the OPTICSxi algorithm, under three initial values for the key parameters ξ (drop in sample density) and *minpts* (number of minimum points): a) low ξ = 0.005, high *minpts* = 8% sample size; b) intermediate ξ = 0.025, intermediate *minpts* = 4% sample size; and c) high ξ = 0.050, low *minpts* = 2%. Black lines represent a repertoire of a photo-identified group of whales, red lines represent the mean discovery curve and red shades represent the standard errors for the mean curve.

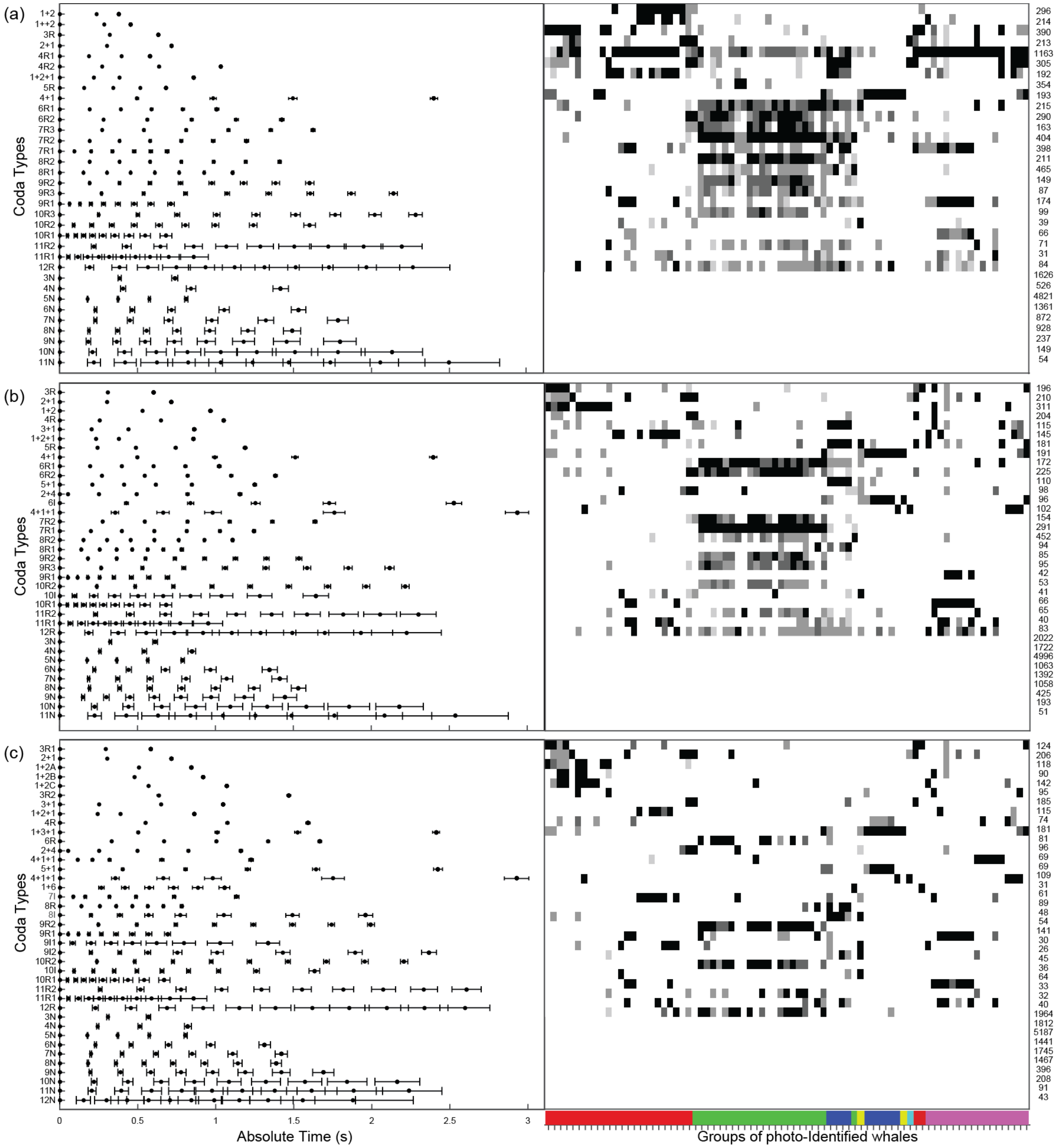

Figure S4. Codas classified into discrete types according to rhythm, produced by photo-identified groups of sperm whales across the Eastern Tropical Pacific. Coda types up to 12 clicks (y-axis) were based on absolute time (x-axis) between inter-click intervals (ICIs). Dots represent mean

time of each click in the coda, and whiskers represent 95% Confidence Intervals around the mean time. Coda types were defined by the OPTICSxi algorithm, under three alternative initial parameterization, varying contrast parameter ξ (threshold in reachability distance drop, establishing the relative decrease in density within clusters) and *minpts* (defines the minimum number of samples that can be considered a cluster) but fixing ξ (maximum reachability distance within which clusters will be searched for): (a) ξ = 0.025, *minpts* ~ 4% of sample size; (b) ξ = 0.005, *minpts* = about 8% of sample size; (c) ξ = 0.05, *minpts* ~ 2% of sample size. Coda type labels (y-axis) were based on the rhythm, where the first number indicates number of clicks, "R" indicates regularly spaced clicks; "I" indicates increasing click intervals along the coda; "+" indicates extended interval between clicks; and the sequential number or letters distinguishes between coda with same length and rhythm but of increasing duration (e.g. 9R1, 9R2; 2+1A, 2+1B). "N" indicate the coda samples regarded as 'noise' by the OPTICS algorithm (note low accuracy, i.e. large 95% confidence intervals) and so discarded from the categorical analyses. The matrices illustrate coda types (rows) for each photo-identified group (columns). Shades of grey indicate the frequency of occurrence of coda types in a given group repertoire (x>10%: black; 5%<x<=10%: dark grey; 1%<x <=5%: grey; 0<x<=1%: light grey; 0: white). Color code for photo-identified groups follow original clan partition in [2] and in the figure 3 (main text). Numbers on the right indicate the total codas per type used in the categorical analysis. Note the high number of coda samples discarded as noise ("N"). Overall, there were some variations across the three parameterization schemes. For instance, the number of coda types would be 25 for the restrictive (a) or 29 for the permissive (c) parameterization schemes, mainly because some 3- and 6-click codas were merged into the same or split into more types. The intermediate parameterization (b) described more clearly the clan segregation based on the continuous analysis data.

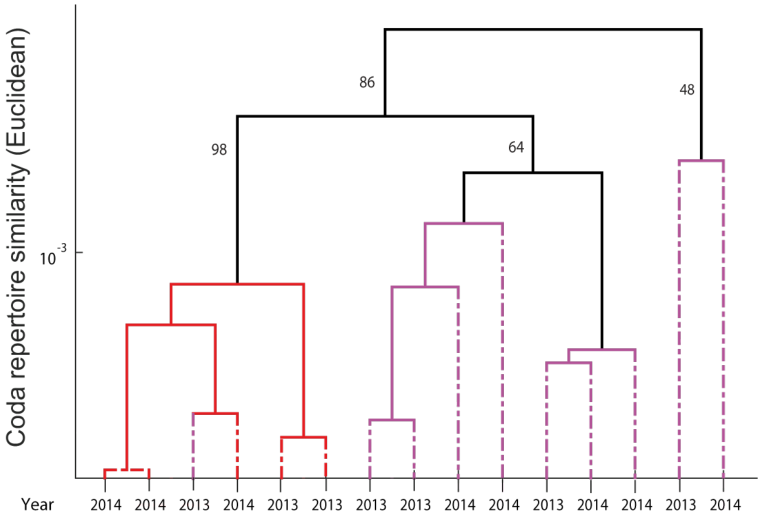

Figure S5. Coda repertoire similarity among only the groups of sperm whales recorded off the Galápagos Islands in 2013 and 2014. The hierarchical clustering dendrogram (average linkage, CCC=0.89) depicts the multivariate similarity (Euclidean distances on absolute inter-click intervals) among coda repertoires of groups of sperm whales (branches). Branches are colored by clans defined by the hierarchical clustering analysis using the entire data set (figure 3a; as in [2]). Numbers besides the clustering branches indicated the number of times (out of 100) they were replicated by in bootstrap analyses; numbers underneath are the year of recording.

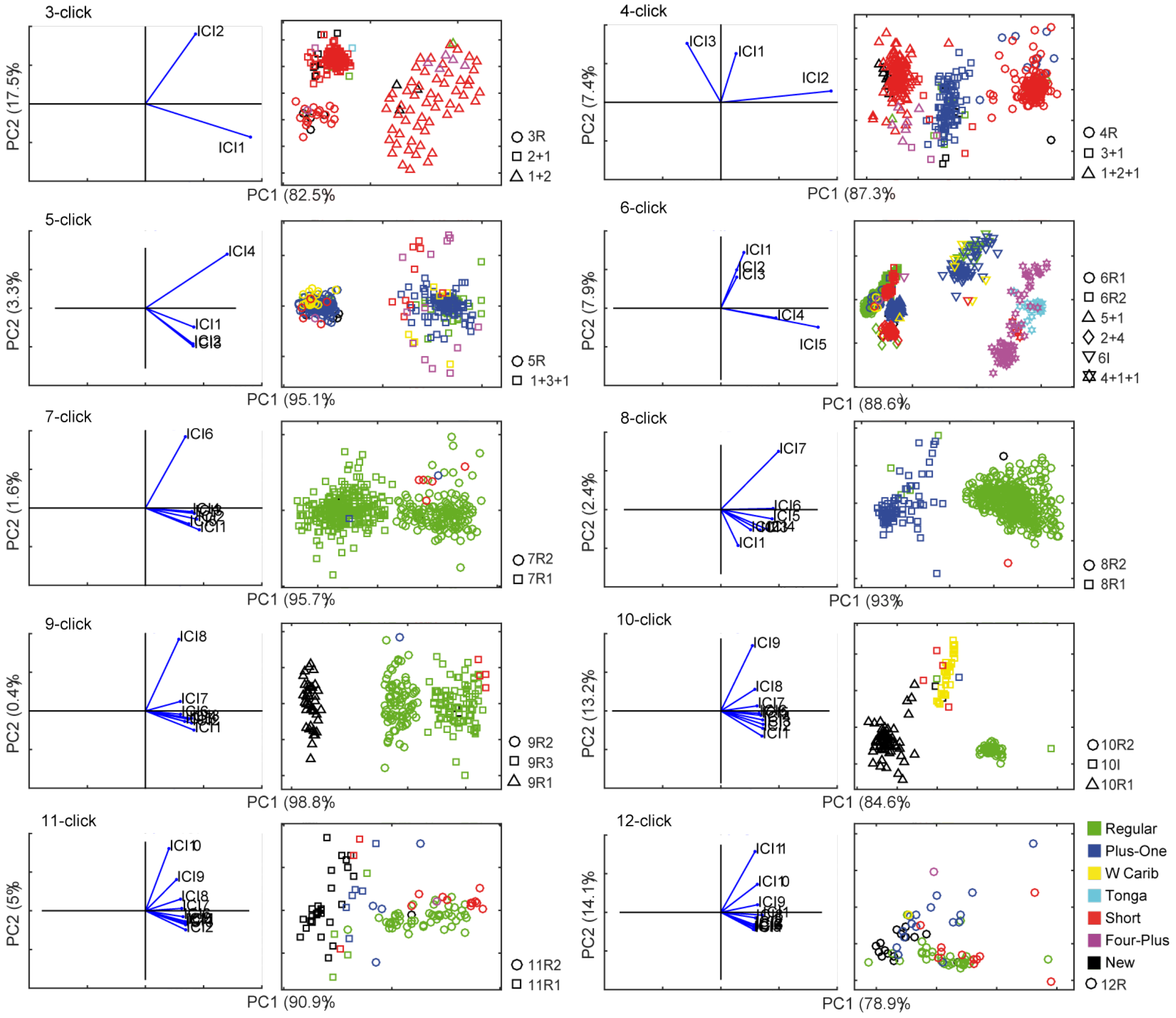

Figure S6. Coda types used by vocal clans of the sperm whales. Principal component analysis was used to reduce the dimensionality of the raw absolute inter-click intervals (ICI) for each coda length (number of clicks) for better visualization of the clusters of codas classified by OPTICSxi into categorical types (figure S2, represented by icon shape) using the intermediate parameterization scheme (figures 3b, S2, S4b). Coda samples are colored by clans, a categorical variable defined by hierarchical clustering analysis (figure 3a; as in [2]). Loading plots indicate the direction of variation according to each ICI. The amount of variance explained by the first two Principal Components (PC) is given at the axes. The new data set recorded off Galápagos in 2013 and 2014 is marked in black. For clarity, all coda samples regarded as 'noise' by the

OPTICSxi algorithm (figure S4) were not plotted. Overall, coda types (here, defined by distinct clusters) were usually defined by the presence/absence of extended pauses ('+'), as suggested by the direction of loadings of the longer ICIs. Note that clans shared some coda types (e.g. 5R, 11R, 12R) but also produced coda types which were nearly exclusive of their own clan. For instance, the *Regular* clan mainly produced regularly-spaced codas from 6 to 12 clicks (e.g. 6-12R); the *Short* clan mainly produced codas with 3 to 5 clicks; *Plus-One* groups produced mainly short codas with an extended pause before the final click (e.g. 3+1, 1+3+1, 5+1, 4+1+1); *Four-Plus* groups produced codas with four regular clicks (e.g. 4R, 4+1+1, 2+4). These characteristic codas used more frequently by specific clans (figure 3b) appears to have driven the clan partitions in the dendrogram presented in the main text (figure 3a).

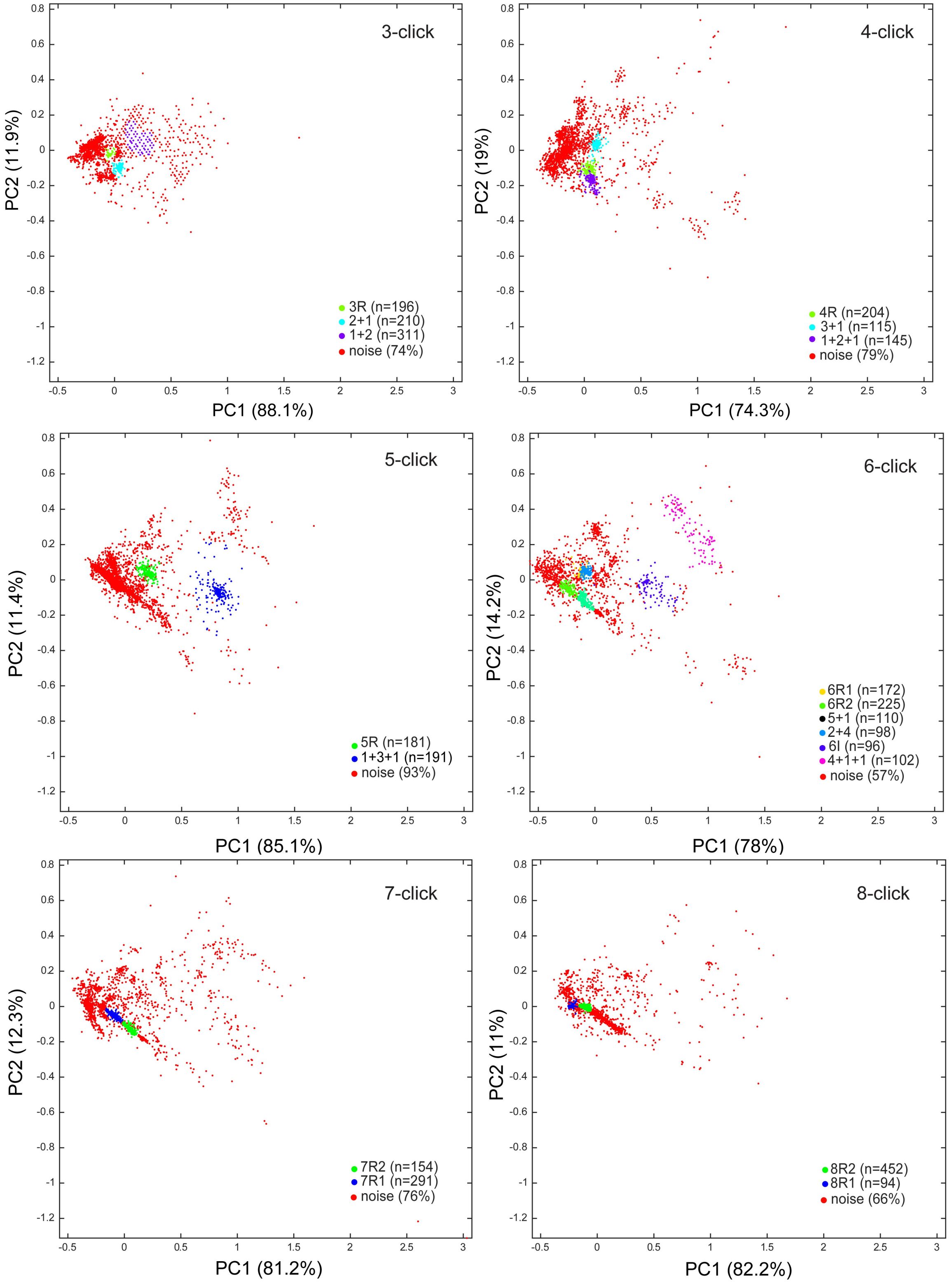

3-click
PC1 (88.1%)
PC2 (11.9%)
3R (n=196)
2+1 (n=210)
1+2 (n=311)
noise (74%)
4-click
PC1 (74.3%)
PC2 (19%)
4R (n=204)
3+1 (n=115)
1+2+1 (n=145)
noise (79%)
5-click
PC1 (85.1%)
PC2 (11.4%)
5R (n=181)
1+3+1 (n=191)
noise (93%)
6-click
PC1 (78%)
PC2 (14.2%)
6R1 (n=172)
6R2 (n=225)
5+1 (n=110)
2+4 (n=98)
6I (n=96)
4+1+1 (n=102)
noise (57%)
7-click
PC1 (81.2%)
PC2 (12.3%)
7R2 (n=154)
7R1 (n=291)
noise (76%)
8-click
PC1 (82.2%)
PC2 (11%)
8R2 (n=452)
8R1 (n=94)
noise (66%)

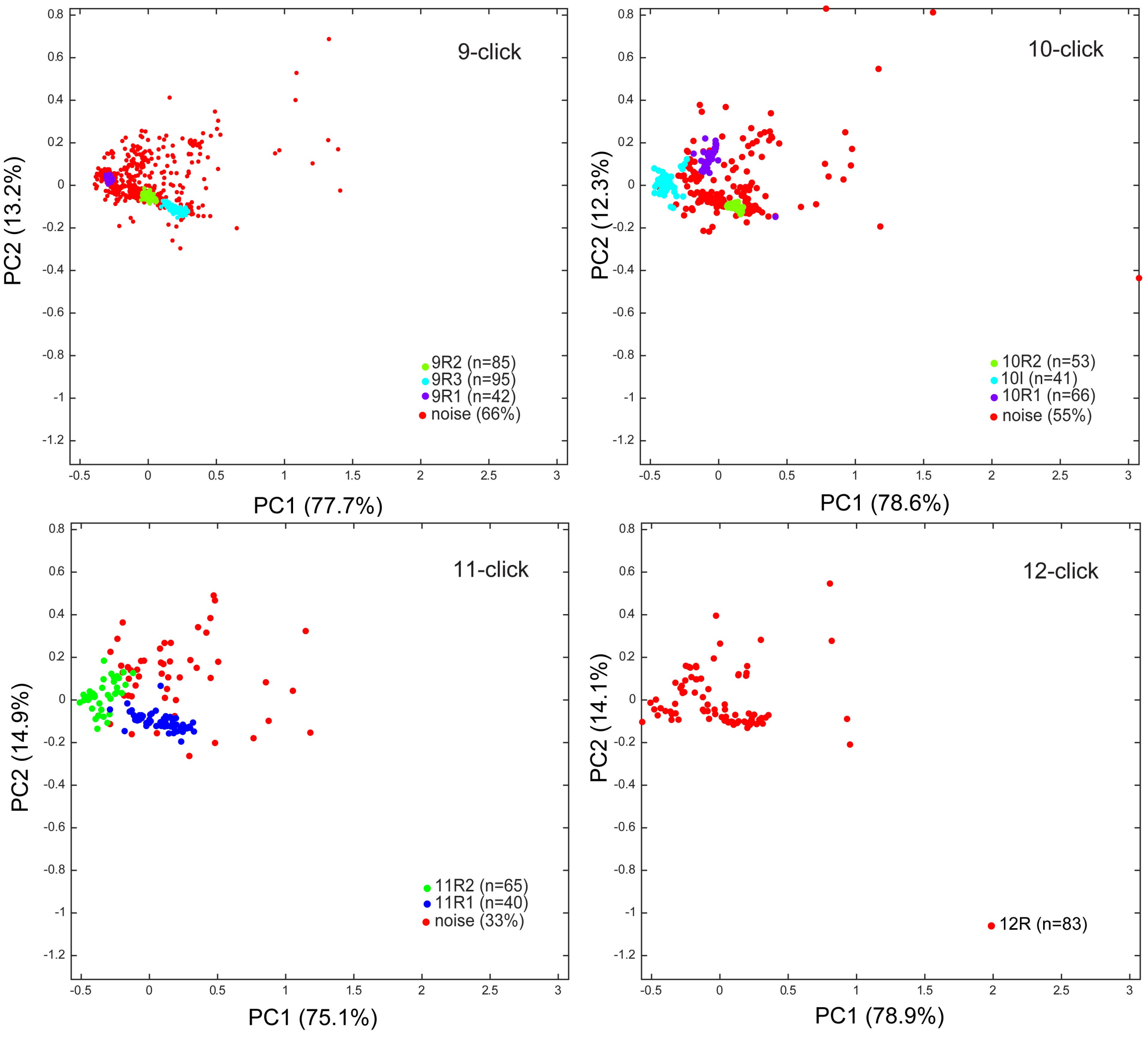

Figure S7. All codas sampled from sperm whales in the Eastern Tropical Pacific as categorized by OPTICSxi including unclassified samples ('noise'). Principal component analysis on the inter-click intervals (ICI) for each coda length (number of clicks) was used here to reduce the dimensionality of the data for better visualization and interpretation. The amount of variance explained by the first two principal components (PC) is given at the axes. Codas are colored by type, as defined by the OPTICSxi algorithm using the intermediate parameterization scheme (figure S4b). Samples sizes by type and proportion of unclassified samples ('noise') by coda length are presented. Note our classification analysis was conservative and discarded a large

portion of the samples regarded as 'noise' by the OPTICSxi algorithm. This creates a scenario in which we only define new coda types when there exists many, highly similar codas.

Supplementary Methods 1: Acoustic recordings

Recordings of sperm whale acoustic repertoires were made when whales were socializing or resting at the surface, and at the beginning of foraging dives [4], using a variety of hydrophone arrays (frequency responses: 6 Hz–10 kHz, ±3 dB; 1–10 kHz, ±3 dB) connected to different recorders and amplifiers (details in Rendell & Whitehead 2003a). In 2013 and 2014, we switched to a custom-built 100m long towed hydrophone array consisting of two Benthos AQ-4 elements (frequency response of 0.1-30kHz) spaced by 3m connected via Magrec HP02 preamplifiers to a Magrec HP27ST amplifying and conditioning box which imposed an analogue high-pass filter at 1kHz or less, and recorded using a laptop PC running PAMGUARD software [5], sampling at 96kHz. To analyze codas, we only use the temporal patterning of their clicks; thus the variation in the sampling or frequency responses of the recording systems used does not impact our analysis.

Supplementary Methods 2: Permutation tests for differences between group repertoires

All coda recordings on a given day from a given group represented a single repertoire, and repertoires from different days were treated as replicates of a group's repertoire [3]. To test for differences in repertoire between groups, we tested the null hypothesis that the coda repertoire similarity between recordings of the same group on two different days (same group, different days: SGDD) is the same as that between recordings of different groups on different days (different groups, different days: DGDD) [3]. We performed Mantel tests (1,000 permutations, Spearman correlation) [6] to test correlations between matrices of multivariate similarity (see below) between sets of codas recorded on pairs of days and a binary matrix in which SGDD=1

and DGDD=0. If different groups have different repertoires, then we would expect greater similarity within a group than between different groups, and so significantly positive correlation between these matrices.

Supplementary Methods 3: Details on continuous similarity of coda repertoires

To quantify the similarity between coda repertoires of groups of whales, we used two complementary approaches: continuous and categorical. The continuous approach is classification-free (disregards coda types) and based on the multivariate similarity of two codas with the same number of clicks using the Euclidean distances between their inter-click interval vectors [7]. We quantified the similarity between entire coda repertoires as [2,7]:

$$S_{AB} = \frac{\sum_{i=1}^{n_A} \sum_{\substack{j=1 \\ l_j=l_i}}^{n_B} \frac{b}{b+d_{ij}}}{n_A \cdot n_B} \qquad (1)$$

where S_{AB} is the similarity between repertoires A with n_A codas and repertoire B with n_B codas; l_i and l_j are the number of clicks in coda i from repertoire A and the number of clicks in coda j in repertoire B; b is the basal similarity (b=0.001) representing a very fine comparison at the order of 1 ms (repertoire similarities are in any case generally robust to variation in b) [7]; and d_{ij} is the Euclidean distance between the inter-click interval vectors of codas i and j [2,7]. Codas with different numbers of clicks were assigned a zero multivariate similarity [2,7,8].

Supplementary Methods 4: Details on categorical analysis of coda repertoires

With the categorical approach to analyze coda repertoires, we classified codas into discrete types to illustrate thematic patterning differences in the repertoires among clans of whales. We assigned categorical types based on the distribution of the absolute ICIs using the OPTICSxi hierarchical clustering [9] in the ELKI framework [10]. We ran OPTICSxi on the

absolute inter-click intervals independently for all coda samples of same number of clicks. Long codas (>12 clicks) were rare (0.9% of 17,045 codas recorded, Table S1) and were not considered for the categorical analysis, but used only in the continuous analysis. We then named discrete codas types based on their rhythm and tempo—following previous nomenclature [2,11]: first number indicates number of clicks, "R" indicates regularly spaced clicks; "I" indicates increasing click intervals; "+" indicates extended interval; and the last number distinguishes between two similar coda types.

OPTICS is a density-based algorithm that orders samples linearly according to their distances apart in the multivariate space; distances are used to calculate "reachability" between samples and then define clusters. OPTICS defines clusters based on the following parameters: the contrast parameter ξ defines the threshold in reachability distance drop, establishing the relative decrease in density between versus within clusters; *minpts* defines the minimum number of samples that can be considered a cluster (details in [9]). The key advantage of OPTICSxi relative to other clustering algorithms is the ability to distinguish clustered samples in multivariate space (i.e. codas with low reachability, or distance in the ordered list) from sparser, outlier samples (i.e. codas with high reachability, located far away from dense clusters). The latter are regarded as 'noise' instead of being forced into a cluster, as in other clustering algorithms (e.g. k-means) [2,7,11]. Therefore, the clusters defined by OPTICSxi (here, coda types) are conservative in membership, since only coda samples with high similarity are considered and any uncertain sample (i.e. the 'noise', with low similarity, between clusters) is discarded. OPTICS density-based algorithm is more successful and accurate in identifying natural clusters in large datasets [12], and so is superior to the k-means algorithm previously used to define coda types [2,7,11] that attempted to classify every sample into a coda type, among other clear limitations [7]. We emphasize that here we used coda classification for descriptive purposes: to illustrate the thematic

patterning of codas driving clan segregation given by the continuous analysis that includes all codas.

There are no rules of thumb to define the OPTICSxi initial parameterization. Therefore, we then ran a sensitivity analysis to find parsimonious parameter values. We first ran the classification algorithm with two extreme conditions: with low drop in sample density and high number of minimum points (low $\xi = 0.005$, high *minpts* = 8% sample size); and high drop in sample density and low minimum points ($\xi = 0.050$, *minpts* = 2%). The two extreme conditions yielded slightly different coda type classifications (figure S3). The former produced many coda types (larger clusters were typically split into many small ones) and the latter produced only a few (visually discrete clusters were not split). Therefore, for our final analyses we used intermediate values for ξ and *minpts*.

For the final classification of codas, we fixed the ξ parameter across all independent analyses of codas of different lengths (i.e. number of clicks), but we adjusted the *minpts* parameter to the corresponding sample sizes. We used $\xi = 0.025$ (i.e. 2.5% drop in sample density defined a new cluster), so considered that the differences between coda types of different lengths are the same [3]. We adjusted *minpts* according to the sample size of each coda length to be around 4% because shorter codas are usually more numerous than longer ones. In all cases, we were very conservative and considered a coda type only the terminal branches in the hierarchical classification discarding all sample that was not in the core of the clusters.

Supplementary Methods 5: Social level definitions

Sperm whale societies contain multiple social levels [4,13]. The fundamental one is the nearly-permanent *social unit*, defined as sets of individuals that live and move together for long periods, from several months to several years [14]. Social units form temporary *groups* among themselves, defining sets of animals that move together in a coordinated manner for periods of

few hours to few days [4]. These groups are formed among social units of the same *vocal clan* [2], defined by sets of social units with high similarity in their coda repertoires [2].

To delineate a social unit, long-term photo-identification data is therefore required (see methodological details in [14]). While this was possible for previous years off the Galápagos [14], there were not enough data available to reliably delineate social units across the Eastern Pacific (see [2]), neither off Galápagos in 2013 and 2014. Therefore, our analyses focused on acoustic repertoires of groups of sperm whales photo-identified together [2]. Social units are known to form these briefer groupings with one another [4], thus in our analysis we may have recorded the acoustic repertoire of more than one social unit. However, this would not affect our analysis because social units are known to group only with other units of the same clan [2].

Supplementary References

[1] Whitehead H, Coakes A, Jaquet N, Lusseau S. 2008 Movements of sperm whales in the tropical Pacific. *Mar. Ecol. Prog. Ser.* **361**, 291-300.

[2] Rendell L, Whitehead H. 2003 Vocal clans in sperm whales (*Physeter macrocephalus*). *Proc. R. Soc. B* **270**, 225-231. (doi: 10.1098/rspb.2002.2239)

[3] Gero S, Whitehead H, Rendell L. 2016 Individual, unit, and vocal clan level identity cues in sperm whale codas. *R. Soc. Open Sci.* **3**, 150372. (doi: http://dx.doi.org/10.1098/rsos.150372)

[4] Whitehead H. 2003 *Sperm whale societies: Social evolution in the ocean*. Chicago, IL: University of Chicago Press.

[5] Gillespie D, Mellinger DK, Gordon J, Mclaren D, Redmond P, McHugh R, Trinder PW, Deng XY, Thode A. 2009 PAMGUARD: Semiautomated, open source software for real-time acoustic detection and localisation of cetaceans. *J. Acoust. Soc. Am.* 125, 2547 (doi: http://dx.doi.org/10.1121/1.4808713)

[6] Schnell GD, Watt DJ, Douglas ME. 1985 Statistical comparison of proximity matrices: applications in animal behaviour. *An. Behav*. **33**, 239–253.

[7] Rendell L, Whitehead H. 2003 Comparing repertoires of sperm whale codas: A multiple methods approach. *Bioacoustics* **14**, 61–81.

[8] Antunes R, Schulz T, Gero S, Whitehead H, Gordon J, Rendell L. 2011 Individually distinctive acoustic features in sperm whale codas. *An. Behav.* **81**, 723-730. (doi:10.1016/j.anbehav.2010.12.019)

[9] Ankerst M, Breunig MM, Kriegel H-P, Sander J. 1999 OPTICS: ordering points to identify the clustering structure. *Proc. SIGMOD'99 Intl. Conf. Manag. Data* **28**, 49–60. (doi:10.1145/304181.304187)

[10] Achtert E, Kriegel HP, Schubert E, Zimek A. 2013 Interactive data mining with 3D-parallel-coordinate-trees. Proc. *2013 ACM SIGMOD Int. Conf. Manag. Data* 1009–1012. (doi:10.1145/2463676.2463696)

[11] Weilgart L, Whitehead H. 1997 Group-specific dialects and geographical variation in coda repertoire in South Pacific sperm whales. *Behav. Ecol. Sociobiol.* **40**, 277-285.

[12] Nanni M, Pedreschi D. 2006 Time-focused clustering of trajectories of moving objects. *J. Intell. Inf. Syst.* **27**, 267–289. (doi:10.1007/s10844-006-9953-7)

[13] Whitehead H, Antunes R, Gero S, Wong SN, Engelhaupt D, Rendell L. 2012 Multilevel societies of female sperm whales (*Physeter macrocephalus*) in the Atlantic and Pacific: why are they so different? *Intl. J. Primatol*. **33**, 1142-1164. (doi: 10.1007/s10764-012-9598-z)

[14] Christal J, Whitehead H, Lettevall E. 1998 Sperm whale social units: variation and change. *Can. J. Zool.* **76**, 1431-1440.
